# Correlation of SUV_max_ and Apparent Diffusion Coefficient Values Detected by Ga-68 PSMA PET/MRI in Primary Prostate Lesions and Their Significance in Lymph Node Metastasis: Preliminary Results of an On-going Study

**DOI:** 10.4274/mirt.galenos.2019.63825

**Published:** 2019-09-06

**Authors:** Lebriz Uslu-Beşli, Barış Bakır, Sertaç Asa, Ekrem Güner, Çetin Demirdağ, Onur Erdem Şahin, Emre Karayel, Muhammet Sait Sağer, Haluk Burçak Sayman, Kerim Sönmezoğlu

**Affiliations:** 1 Istanbul University-Cerrahpasa Faculty of Medicine, Department of Nuclear Medicine, İstanbul, Turkey; 2 Istanbul University İstanbul Faculty of Medicine, Department of Radiology, İstanbul, Turkey; 3 University of Health Sciences, Bakırköy Dr. Sadi Konuk Training and Research Hospital, Clinic of Urology, İstanbul, Turkey; 4 İstanbul University-Cerrahpasa Faculty of Medicine, Department of Urology, İstanbul, Turkey

**Keywords:** Gallium-68, prostate specific membrane antigen, positron emission tomography/magnetic resonance imaging, multiparametric prostate magnetic resonance imaging, prostate cancer, lymph node metastasis

## Abstract

**Objectives:**

Gallium-68 (Ga-68) prostate specific membrane antigen (PSMA) positron emission tomography (PET) has been shown to be more accurate than multiparametric prostate magnetic resonance imaging (MRI) in detection of primary prostate lesions. Using hybrid PET/MRI we aim to detect the correlation between SUV_max_ and apparent diffusion coefficient (ADC) in primary prostate lesions and to assess their prognostic value in detection of lymph node (LN) metastasis.

**Methods:**

Twenty-six patients, who were diagnosed as having prostate cancer with biopsy and underwent Ga-68 PSMA PET/MRI together with biparametric prostate MRI (bpMRI) were included. SUV_max_, SUV_mean_ and ADC were recorded for index lesions drawing a region of interest (ROI) of 1 cm^2^ around the pixel with the highest SUV_max_ (ROI-1) and another ROI following borders of prostate tumor detected by bpMRI (ROI-2). Presence of LN metastasis was recorded according to PSMA PET/MRI.

**Results:**

SUV_max_ was inversely correlated with ADC (ROI-1: p=0.010; ROI-2: p=0.017 for b=800). SUV_max_ and SUV_means_ were both higher in patients with LN metastasis and ADC was lower in patients with LN metastasis for ROI-1. SUV_max_ cut-off value of 19.8 for ROI-1 and 20.9 for ROI-2 had sensitivity and specificity of 77.8% and 76.5%, respectively for detection of LN metastasis, whereas ADC (b=800) cut-off value of 0.92x10^-3^ mm^2^/s had sensitivity and specificity of 87.5% and 76.5%, respectively. SUV_max_/ADC (b=800) ratio increased the sensitivity and specificity to 100% and 82.4%, respectively.

**Conclusion:**

SUV and ADC values are inversely correlated in primary prostate lesions and the combined use of both values increases the diagnostic accuracy of hybrid PET/MRI in the detection of primary prostate lesions.

## Abstract (tr)

**Amaç:**

Galyum-68 (Ga-68) prostat spesifik membran antijeni (PSMA) pozitron emisyon tomografinin (PET) primer prostat lezyonunu saptamada multiparametrik prostat manyetik rezonans görüntülemeden (MRG) daha doğru olduğu gösterilmiştir. Hibrit PET/MRG kullanarak primer prostat lezyonlarında SUV_maks_ ve görünür difüzyon katsayısı (ADC) arasındaki korelasyonu tespit etmeyi ve lenf nodu (LN) metastazı tespitinde prognostik değerlerini saptamayı amaçlıyoruz.

**Yöntem:**

Biyopsi ile prostat kanseri tanısı almış, biparametrik prostat MRG (bpMRG) ile birlikte Ga-68 PSMA PET/MRG yapılmış 26 hasta çalışmaya alındı. İndeks lezyonların SUV_maks_, SUV_ortalama_ ve ADC değerleri, hem en yüksek SUV_maks_ olan piksel etrafında 1 cm^2^ çapında ilgi alanı (ROI) çizerek (ROI-1) hem de bpMRI tarafından tespit edilen prostat tümörünün sınırlarını takip eden bir başka ROI çizerek (ROI-2) kaydedildi. LN metastazı varlığı PSMA PET/MRG’ye göre belirlendi.

**Bulgular:**

SUV_maks_ ve ADC arasında ters korelasyon saptandı (b=800 için ROI-1: p=0,010; ROI-2: p=0,017). SUV_maks_ ve SUV_ortalama_ değerleri LN metastazlı hastalarda daha yüksek bulundu ve ADC değeri ROI-1 için LN metastazlı hastalarda daha düşüktü. SUV_maks_ sınır değeri ROI-1 için 19,8 ve ROI-2 için 20,9 alındığında LN metastazının saptanmasında duyarlılık ve özgüllük sırasıyla %77,8 ve %76,5 olarak bulundu. Buna karşılık ADC (b=800) sınır değeri 0,92x10^-3^ mm^2^/s alındığında duyarlılık ve özgüllük sırasıyla %87,5 ve %76,5 olarak saptandı. SUV_maks_/ADC (b=800) oranı, duyarlılığı ve özgüllüğü sırasıyla %100 ve %82,4’e yükseltti.

**Sonuç:**

SUV ve ADC değerleri primer prostat lezyonlarında ters korelasyon gösterir ve her iki değerin birlikte kullanımı primer prostat lezyonlarının tespitinde hibrit PET/MRG’nin tanısal doğruluğunu arttırır.

## Introduction

Prostate cancer is the second most common diagnosed cancer in men and the fifth leading cause of cancer-related death worldwide (1). Death rates are lower in developed countries, due to early detection of the disease and improved treatment methods (1). Prostate specific antigen (PSA) is a glycoprotein produced by prostate cells and though not specific for prostate cancer, elevated PSA values detected by PSA screening was shown to aid in early diagnosis of prostate cancer, thus decrease prostate cancer-related death rates (2,3,4).

The screening for prostate cancer is generally made by serum PSA level measurement together with digital rectal examination (DRE). Prostate 12-core needle biopsy under transrectal ultrasonography guidance (TRUS-biopsy) is the most common method used for diagnosis of prostate cancer (5). Multiparametric prostate magnetic resonance imaging (mpMRI) has been introduced as a novel imaging approach for diagnosis and localization of primary prostate lesions (6). MpMRI guided prostate biopsy was shown to be more accurate than conventional TRUS-biopsy (7). Therefore, although mpMRI is not routinely recommended as a screening tool for detection of prostate cancer, it is recommended for patients with elevated PSA values despite negative TRUS-biopsy (8,9,10).

The most common sites for metastasis in prostate cancer are bones (84%), distant lymph nodes (LN) (10.6%), liver (10.2%) and thorax (9.1%) (11). However, magnetic resonance imaging (MRI) alone has limited value in detection of LN and distant organ metastasis.

Prostate specific membrane antigen (PSMA), which functions on cell membrane as glutamate carboxypeptidase-2 or folate hydrolase, was shown to be over-expressed in prostate cancer cells (12), which led to the introduction of Ga-68 labeled urea-based PSMA inhibitor (Ga-68-PSMA-HBED-CC) as a novel positron emission tomography (PET) tracer used for staging of patients with prostate cancer with high accuracy, for detection of LN and organ metastasis, as well as for detection of residual or recurrent local disease (13). PSMA overexpression in prostate cancer cells was shown to be associated with higher prostate cancer grade, resulting in higher incidence of metastasis and castration resistance (14). Similarly, apparent diffusion coefficient (ADC) value obtained from diffusion-weighted imaging (DWI) component of mpMRI was shown to be inversely correlated with Gleason score and was reported to provide quantitative information on tumor characteristics and aggressiveness (15). Hybrid PET/MRI systems have also been shown to be more accurate than mpMRI in terms of detecting primary prostate lesions (16,17).

The aim of our study is to detect the correlation between maximum and mean standardized uptake value (SUV_max_ and SUV_mean_) and ADC values of primary prostate lesions and to assess the prognostic value of SUV_max_ and ADC in terms of detecting LN metastasis.

## Materials and Methods

This retrospective study was approved by İstanbul University Clinical Research Ethics Committee (14/01/2019-6927) and conducted between May 2017 and April 2018. All procedures performed in this study involving human participants were in accordance with the ethical standards of the Institutional and/or National Research Committee and with the 1964 Helsinki Declaration and its later amendments or comparable ethical standards.

### Study Population

Twenty-six patients, with a mean age of 67.5±7.0 years (median age: 67.5, range: 50-83 years), who were diagnosed as having prostate cancer using TRUS-biopsy and underwent whole body Ga-68 PSMA PET/computerized tomography (PET/CT) or PET/MRI together with prostate PET/MRI including biparametric-MRI (bpMRI) sequences were included in our retrospective analysis. The patients had elevated serum PSA values (mean: 65.2±199.6 ng/mL, median: 21.4 ng/mL, range: 5.4-934 ng/mL) and they did not receive any previous treatment or did not undergo any operation related with prostate cancer or with benign prostate hyperplasia previously. Patient characteristics are given in Table 1.

### Imaging

For Ga-68 PSMA PET imaging, all patients were injected Ga-68-PSMA-HBED-CC with a mean activity of 255.3±77.7 MBq (6.9±2.1 mCi), intravenously. Radiolabeling procedure was performed using a fully automated radiopharmaceutical synthesis device based on a modular concept (Eckert & Ziegler Eurotope, Berlin, Germany) as described previously by Kabasakal et al. (18).

All PET/MRI images were acquired using an integrated 3 Tesla - PET/MRI scanner (GE Signa PET/MRI, GE Healthcare, Waukesha, Wisconsin, USA). Prostate PET/MRI including bpMRI was acquired at mean 104.9±43.6 minutes post-injection including an initial localizer scan, a 3D dual-echo fast spoiled gradient recalled echo liver-accelerated volume acquisition sequence (LAVA-FLEX) for MRI based attenuation correction (MRAC), followed by a high-resolution axial T1-weighted (T1W) 3D LAVA-FLEX sequence, T2-weighted (T2W) periodically rotated overlapping parallel lines with enhanced reconstruction (PROPELLER) technique at 3-planes (axial, sagittal and coronal) and field of view optimized and constrained undistorted single shot (FOCUS) DWI (b values: 50-400-800 and 50-1400) and ADC mapping. PET emission scan was recorded together with MRI sequences and acquisition time per bed position was 3.5 min. PET attenuation correction was performed using vendor-based algorithm including MRAC data and atlas-based attenuation correction map.

A total of 10 patients had a whole-body PET/MRI at mean 87.5±20.3 minutes post-injection in the caudo-cranial direction from mid-thigh to vertex, including an initial localizer scan, 3D LAVA-FLEX for MRAC, high-resolution axial T1W 3D LAVA-FLEX sequence, coronal T2W short-tau inversion recovery (STIR), axial DWI (b values: 50-1000) and ADC mapping.

A total of 16 patients had whole-body Ga-68 PSMA PET/CT images acquired prior prostate PET/MRI using an integrated PET/CT scanner (Siemens Biograph 6, Knoxville, TN, USA or GE Discovery 710, Waukesha, WI, USA) at 71.6±14.4 minutes post-injection. An initial CT topogram was followed by a CT transmission scan and an emission PET scan in the caudo-cranial direction from mid-thigh to vertex. Imaging parameters for transmission CT scan were as follows: Low tube current (130 kVp 48-76 mAs), slice thickness of 4.0 mm, gantry rotation time of 0.6 s and collimator width of 6x3 mm. PET emission scan was acquired at 2-4 min per bed position (GE Discovery PET/CT: 2 min/bed, Siemens Biography 6 PET/CT: 4 min/bed) at caudo-cranial direction. Iterative image reconstruction method using CT transmission images were utilized for attenuation correction. All patients were asked to empty bladder before initiation of whole-body PET/CT or PET/MRI as well as prostate PET/MRI acquisition to minimize bladder activity.

### Image Analysis

All whole-body PET images (PET/CT and PET/MRI) were reviewed and analyzed by two nuclear medicine physicians (LUB and SA) together and all prostate PET/MRI images including bpMRI sequences were reviewed together with a radiologist (BB) and a nuclear medicine physician (LUB) together using vendor-based work station (GE AW Volume Share 7, GE Medical Systems, Buc, France). Localization and extension of the primary tumor in the prostate gland was recorded on a prostate scheme for both PET and MRI data separately. SUV_max_ and mean ADC (ADC) were measured drawing region of interest (ROI) 1 cm^2 ^around the pixel with the highest SUVmax in the prostate tumor (ROI-1) and another ROI following the borders of prostate tumor (ROI-2) detected by bpMRI. Whole-body PET images were used to detect presence of LN and organ metastasis.

### Statistical Analysis

Statistical analysis was performed using SPSS software version 21.0 (IBM Corp., Armonk, New York, USA) and the level of significance was taken as p value less than 0.05. Pearson correlation analysis was performed to observe the relationship between SUV and ADC values. Mann-Whitney U test was performed to analyze the relationship between LN metastasis status and SUV and ADC values. Receiver operating characteristic (ROC) curve analysis was calculated to assess the ability to discriminate the LN metastasis status based on SUV and ADC values.

## Results

SUV_max_, SUV_mean_ and ADC values (for both b=1400 and b=800) obtained from ROI-1 and ROI-2 of the prostate lesion were given in Table 2.

**Figure 1:**
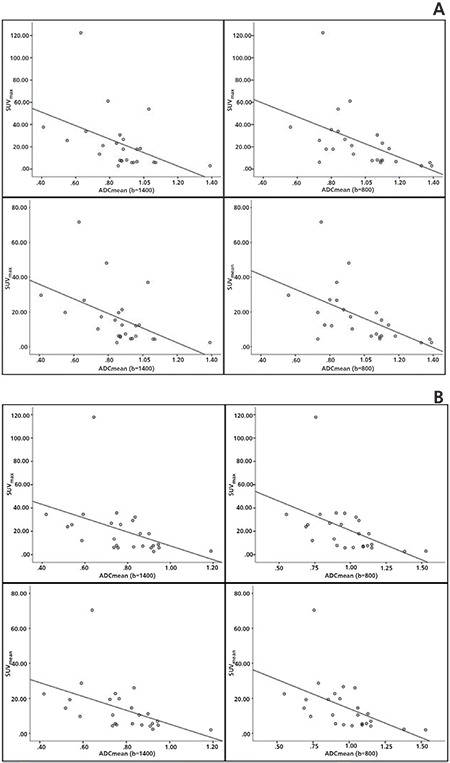
Both SUV_max_ and SUV_mean_ values were found to be inversely correlated with ADC values (both for b=1400 and b=800) for ROI-1 (A) and ROI-2 (B) SUV_max_: Maximum standardized uptake value, SUV_mean_: Mean standardized uptake value, ADC_mean_: Mean apparent diffusion coefficient value, ROI: Region of interest

For both ROI-1 and ROI-2, SUV_max_ value was inversely correlated with both ADC (b=1400) value (ROI-1: p=0.026, r=-0.444; ROI-2: p=0.032, r=-0.429) and ADC (b=800) value (ROI-1: p=0.010, r=-0.506; ROI-2: p=0.017, r=-0.473) (Table 3). Also, SUV_mean_ value was inversely correlated with both ADC (b=1400) value (ROI-1: p=0.013, r=-0.488; ROI-2: p=0.018, r=-0.468) and ADC (b=800) value (ROI-1: p=0.004, r=-0.553; ROI-2: p=0.009, r=-0.508) for both ROI-1 and ROI-2 (Figure 1A, 1B).

**Figure 2:**
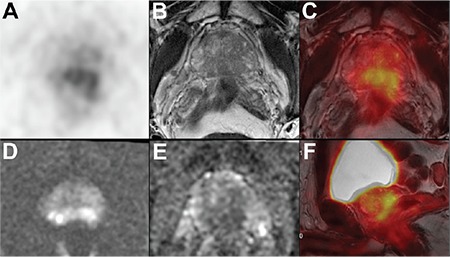
Sixty-nine-year-old patient with Gleason score 4+3 prostate cancer detected by prostate 12-core needle biopsy under transrectal ultrasonography guidance. His serum prostate specific antigen level was 27.0 ng/mL at the time of diagnosis. Axial Ga-68 prostate specific membrane antigen (PSMA) positron emission tomography (PET) (A), axial T2-weighted magnetic resonance imaging (MRI) (B), fused PET/ MRI (C), FOCUS diffusion weighted imaging for b=1400 s/mm^2^ (D), apparent diffusion coefficient (ADC) map for b=800 s/mm^2^ (E) and sagittal fused PET/MRI (F) images exhibited PSMA-positive tumor located at bilateral peripheral zone of the prostate gland (SUV_max_: 6.7, SUV_mean_: 6.1, ADC: 1.18 s/mm^2^ for region of interest (ROI)-1 and SUV_max_: 6.6, SUV_mean_: 5.5, ADC: 1.09 s/mm^2^ for ROI-2). The patient did not have any lymph node or organ metastasis according to the PSMA PET images

**Figure 3:**
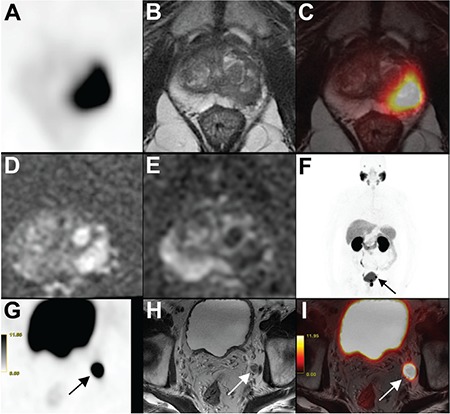
Eighty-three-year-old patient with Gleason score 4+4 prostate cancer detected by prostate 12-core needle biopsy with serum prostate specific antigen level of 5.8 ng/mL at the time of diagnosis. Axial Ga-68 prostate specific membrane antigen (PSMA) positron emission tomography (PET) (A), axial T2-weighted (T2W) magnetic resonance imaging (MRI) (B), fused PET/MRI (C), FOCUS diffusion weighted imaging for b=1400 s/mm^2^ (D) and apparent diffusion coefficient (ADC) map for b=800 s/mm^2^ (E) images showed intense PSMA uptake at the prostate tumor located at left peripheral zone (SUV_max_: 53.9, SUV_mean_: 37.0, ADC: 0.84 s/mm^2^ for region of interest (ROI)-1 and SUV_max_: 32.2, SUV_mean_: 26.0, ADC: 1.04 s/mm^2^ for ROI-2). Metastatic pelvic lymph node with intense PSMA uptake can be seen on maximum intensity projection (F) image, axial PSMA PET (G), axial T2W MRI (H) and fused PET/MRI (I) images (arrows)

SUV_max_ and SUV_mean_ were significantly higher in patients with LN metastasis for both ROI-1 and ROI-2 (ROI-1: p=0.01 and p=0.01; ROI-2: p=0.02 and p=0.01, respectively) (Table 4) (Figures 2, 3). Although ADC values were significantly lower in patients with LN metastasis for ROI-1 (p=0.04 for b=1400 and p=0.02 for b=800), there was no significant difference in terms of ADC values in patients with LN metastasis for ROI-2. The ratios of SUV_max_/ADC and SUV_mean_/ADC for both b values (b=1400 and b=800) were significantly higher in patients with LN metastasis for both ROI-1 and ROI-2 (Table 4).

ROC analysis revealed that SUV_max_ cut-off level of 19.8 for ROI-1 and 20.9 for ROI-2 predicted the presence of LN metastasis with sensitivity of 77.8% and specificity of 76.5% (Table 5). For SUV_mean_, cut-off level of 16.3 for ROI-1 and 10.8 for ROI-2 had sensitivity of 77.8% and 88.9% and specificity of 82.4% and 76.5%, respectively. For ADC (b=800) and ADC (b=1400) cut-off levels of 0.92x10^-3^ mm^2^/s and 0.82x10^-3^ mm^2^/s had sensitivities of 87.5% and 50% and specificities of 76.5% and 82.4%, respectively in prediction of LN metastasis. When SUV/ADC ratios were taken for both SUV_max_ and SUV_mean_ values as well as for both ADC values; sensitivity and specificity increased to 100% and 82.4%, respectively for ROI-1 and to 87.5% and 82.4%, respectively for ROI-2 (Table 5).

## Discussion

MpMRI has been introduced as a novel imaging approach for diagnosis, localization and characterization of primary prostate lesions and has been shown to have a good sensitivity for detecting clinically significant prostate cancer and guiding prostate biopsy (19,20). However, despite its several advantages, mpMRI has also some limitations, including poor detection of low-grade disease, low inter-observer agreement, poor quality images within six weeks after TRUS-biopsy due to residual hemorrhage and inflammation, limited patient cooperation, especially in claustrophobic patients and lower sensitivity in transitional zone tumors (19,21). Ga-68 PSMA PET/CT and PET/MRI, on the other hand were shown to have better sensitivity and higher diagnostic accuracy than mpMRI in the detection of primary prostate cancer, both in index lesions and in cases of multifocal disease (17,22,23,24).

Ga-68 PSMA uptake was shown to be correlated with tumor Gleason score, serum PSA levels, PI-RADS category and DRE findings (25). ADC value obtained by mpMRI was also found to be correlated with Gleason scores (26,27), serum PSA levels (28), molecular markers (29) and was introduced to be a promising tool to monitor therapy response (30,31). To our knowledge, PSMA uptake and ADC values were not compared before using hybrid PET/MRI or PET/CT systems. However, an inverse correlation between PSMA uptake and ADC values in primary prostate tumor is an expected finding according to the current literature.

Hybrid PET/MRI systems provide better anatomical delineation of prostate gland compared to hybrid PET/CT systems due to better soft-tissue resolution of the MRI component, and enable one-stop-shop imaging for prostate cancer patients, including Ga-68 PSMA PET and mpMRI in a single session. Therefore, PET/MRI has more potential to aside misdiagnosis due to physiological or false-positive PSMA uptake in the prostate gland (32,33). Also, simultaneous acquisition of PET and MRI images could provide additional advantages, which are not provided by PET/CT systems. However, to date, there are still limited number of studies on Ga-68 PSMA PET/MRI in evaluation of primary prostate tumor and in its diagnostic accuracy compared to PET/CT or mpMRI.

We found higher SUV_max_ and SUV_mean_ values and lower ADC values in patients with LN metastasis, which may be due to the presence of more aggressive tumor with higher Gleason scores, that were documented to have both higher PSMA uptake and lower ADC values in the literature (26,27,34). Concomitant usage of SUV and ADC parameters by using the ratio of SUV/ADC further increased the sensitivity and specificity of PET/MRI imaging in predicting LN metastasis.

### Study Limitations

The main limitation in our study was the lack of post-operative histopathological result in our cohort and the small sample size. Therefore, we could not compare PSMA uptake with Gleason scores and we had to evaluate the status of LN metastasis only by Ga-68 PSMA PET imaging.

## Conclusion

SUV and ADC values are inversely correlated in primary prostate lesions and the combined use of both values increases the diagnostic accuracy of hybrid PET/MRI in the detection of primary prostate lesions. SUV_max_, SUV_mean_ and ADC detected by Ga-68 PSMA PET/MRI are future promising new prognostic values for detecting LN metastasis in prostate cancer patients.

## Figures and Tables

**Table 1:**
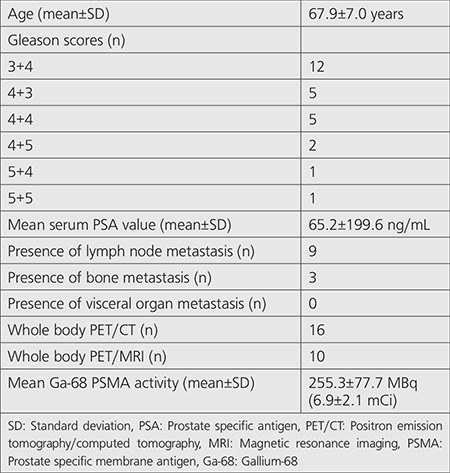
Patient characteristics

**Table 2:**
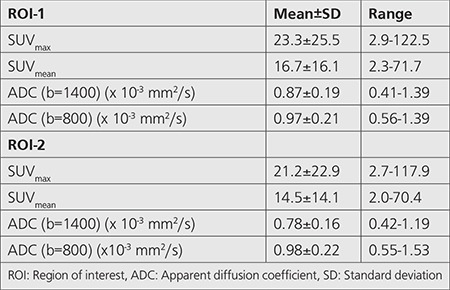
Mean maximum and mean standardized uptake values and apparent diffusion coefficient values of prostate lesions obtained from drawing 2 different region of interests

**Table 3:**
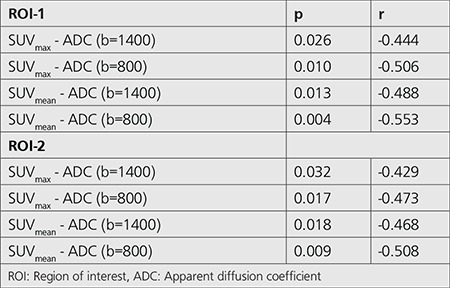
Correlation analysis between maximum and mean standardized uptake value and apparent diffusion coefficient

**Table 4:**
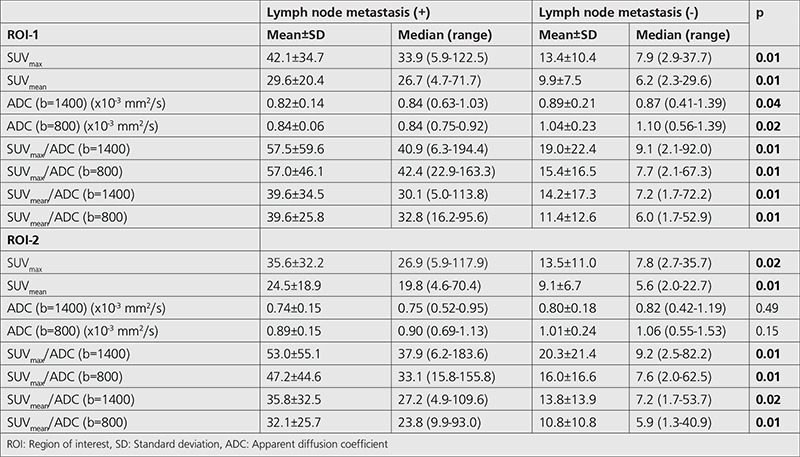
Comparison of standardized uptake value parameters and apparent diffusion coefficient values according to presence of lymph node metastasis

**Table 5:**
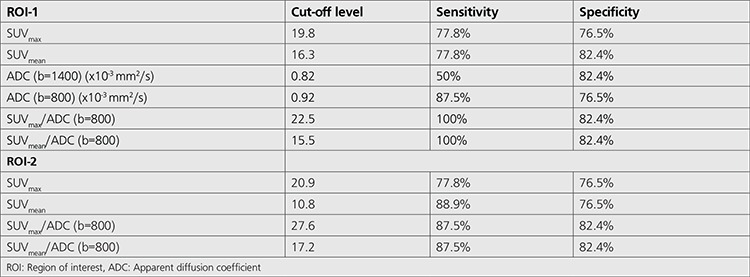
Sensitivity of specificity of different standardized uptake value parameters and apparent diffusion coefficient values in terms of prediction of lymph node metastasis
